# Prolonged Zaleplon Treatment Increases the Expression of Proteins Involved in GABAergic and Glutamatergic Signaling in the Rat Hippocampus

**DOI:** 10.3390/brainsci13121707

**Published:** 2023-12-12

**Authors:** Jelena Martinovic, Janko Samardzic, Marina Zaric Kontic, Sanja Ivkovic, Sanja Dacic, Tamara Major, Milica Radosavljevic, Dubravka Svob Strac

**Affiliations:** 1 Department of Molecular Biology and Endocrinology, VINCA Institute of Nuclear Sciences, National Institute of the Republic of Serbia, University of Belgrade, P.O. Box 522-090, 11000 Belgrade, Serbia; marinazaric@vin.bg.ac.rs (M.Z.K.); sivkovic@vinca.rs (S.I.); 2 Institute of Pharmacology, Clinical Pharmacology and Toxicology, Faculty of Medicine, University of Belgrade, 11000 Belgrade, Serbia; janko.samardzic@med.bg.ac.rs (J.S.); milica.radosavljevic@med.bg.ac.rs (M.R.); 3 Department of General Physiology and Biophysics, Institute of Physiology and Biochemistry “Ivan Djaja”, Faculty of Biology, University of Belgrade, 11000 Belgrade, Serbia; sanjas@bio.bg.ac.rs; 4 Faculty of Pharmacy, University of Belgrade, 11000 Belgrade, Serbia; majtamara@gmail.com; 5 Laboratory for Molecular Neuropsychiatry, Division of Molecular Medicine, Rudjer Boskovic Institute, 10000 Zagreb, Croatia; dsvob@irb.hr

**Keywords:** zaleplon, GABA, glutamate, adenosine, hippocampus, Z drugs, rats

## Abstract

Zaleplon is a positive allosteric modulator of the γ-aminobutyric acid (GABA)_A_ receptor approved for the short-term treatment of insomnia. Previous publications on zaleplon have not addressed the proteins involved in its mechanism of action but have mostly referred to behavioral or pharmacological studies. Since both GABAergic and glutamatergic signaling have been shown to regulate wakefulness and sleep, we examined the effects of prolonged zaleplon treatment (0.625 mg/kg for 5 days) on these systems in the hippocampus of male Wistar rats. Western blot and immunohistochemical analyses showed that the upregulated components of GABAergic signaling (glutamate decarboxylase, vesicular GABA transporter, GABA, and α1 subunit of the GABA_A_ receptor) were accompanied by increased protein levels in the glutamatergic system (vesicular glutamate transporter 1 and NR1, NR2A, and NR2B subunits of N-methyl-d-aspartate receptor). Our results, showing that zaleplon enhances GABA neurotransmission in the hippocampus, were not surprising. However, we found that treatment also increased glutamatergic signaling. This could be the result of the downregulation of adenosine A1 receptors, important modulators of the glutamatergic system. Further studies are needed to investigate the effects of the zaleplon-induced increase in hippocampal glutamatergic neurotransmission and the possible involvement of the adenosine system in zaleplon’s mechanism of action.

## 1. Introduction

Zaleplon treatment (5–20 mg, lasting from a few days to 2 weeks, with a maximum of 4 weeks) is currently indicated for the short-term management of symptoms in patients with initial insomnia [1]. This member of non-benzodiazepine hypnotics, collectively called the Z drugs, is pyrazolopyrimidine, which, due to the quick onset of action and ultrashort half-life (achieving peak plasma concentration and elimination half-life in approximately 1 h), is particularly effective for sleep induction rather than its maintenance.

Zaleplon binds at the interface of the α and γ subunits of the γ-aminobutyric acid (GABA_A_) receptor, acts as a positive allosteric modulator, and causes a conformational change in the receptor’s structure. This modification results in a higher affinity of the receptor binding site for GABA, thus increasing the frequency of the chloride ion channel opening and, consequently, inhibiting brain excitability [2]. To date, most of the literature data on the effects of zaleplon refer to pharmacological and behavioral studies. It was demonstrated that zaleplon has a much higher affinity for the α1-containing subtype than for the α2-, α3-, and α5-containing subtypes of GABA_A_ receptors and that this α1 selectivity may underlie the predominant sedative–hypnotic actions of zaleplon [3]. Moreover, the binding selectivity and pharmacokinetic profile of zaleplon minimize the possibility of side effects such as those produced by benzodiazepines (next-day sedation, dependence, withdrawal). Still, although Z drugs appeared on the market as safe substitutes for benzodiazepines, cases of misuse, abuse, dependence, and death involving these medications have also been reported [1,4]. As for behavioral studies, zaleplon has been reported to have a preferential sedative effect in rats without impairing learning and memory [5]. In addition, Troy [6] and Verster [7], along with their colleagues, have shown, in studies of healthy volunteers receiving a single therapeutic dose of zaleplon, that it is a safe hypnotic that does not impair memory and learning. The lack of association between Z drugs and cognitive impairment may be due, in part, to their affinity for the α1 GABA_A_ subunit rather than the α5 GABA_A_ subunit [8], as it has been speculated that benzodiazepines may increase the risk of cognitive impairment through α5 GABA_A_ in the hippocampus. Zaleplon has also been shown not to impair sleep-dependent synaptic plasticity [2].

A growing body of evidence suggests that the backbone of the sleep−wake regulatory system is largely dependent on the neurotransmitters glutamate and GABA [9]. Through their different distributions, proportions, and discharge profiles, glutamatergic and GABAergic neurons contribute to the generation and shifting of sleep−wake states. This fine-tuning can be achieved by controlling the presynaptic release of the neurotransmitters glutamate and GABA as a result of changes in their metabolism (synthesis or degradation), compartmentalization, release, and recycling. However, it is generally believed that plasticity mechanisms in the brain are primarily mediated by the regulation of glutamate and GABA receptor expression and function [10].

Given that research on zaleplon at the molecular level is sparse, the aim of our study was to examine the expression of synaptic proteins involved in GABAergic and glutamatergic signaling in the hippocampus of male Wistar rats treated with zaleplon for 5 days. The 67 kDa isoform of glutamate decarboxylase (GAD67), an enzyme responsible for the synthesis of GABA from glutamate, the vesicular GABA transporter (VGAT), which mediates the accumulation of GABA into synaptic vesicles, as well as the protein expression of the α1 subunit of the GABA_A_ receptor, which is responsible for the sedative–hypnotic effect of zaleplon, were investigated using the Western blot technique. The GABA content in the hippocampus was analyzed using immunohistochemical staining. In addition, we investigated the zaleplon-induced changes in the protein levels of the vesicular glutamate transporter 1 (vGlut1), which mediates glutamate uptake into synaptic vesicles, and the NR1, NR2A, and NR2B subunits of the N-methyl-d-aspartate (NMDA) glutamate receptor.

Increased expression was found for all the parameters examined. While the results related to the components of the GABAergic signaling system are consistent with the proposed role and mechanism of action of zaleplon as an hypnotic [11], we sought the mechanism explaining the upregulation of the components of the glutamatergic signaling system.

Adenosine is a naturally occurring purine nucleoside that is an important modulator of neuronal activity. It is released by almost all cells and influences the release of neurotransmitters, receptors, and neuronal and glial transporters or controls the function of other neuronal modulators. Once released, adenosine can act via four different types of G protein-coupled receptors from the P1 receptor family, namely, adenosine receptors (A1R, A2_A_R, A2_B_R, A3) [12]. The A1R is widely distributed in the hippocampus and is heterogeneously expressed in neurons and interneurons in a pre- and postsynaptic density as well as in non-neuronal cells [13,14]. Presynaptically, A1Rs reduce neurotransmitter release by the G protein-coupled inhibition of voltage-dependent Ca^2+^ channels (VDCCs) or by inhibition of the Ca^2+^-independent spontaneous release of neurotransmitters. In addition, adenosine A1R inhibits the activity of postsynaptic VDCCs and NMDARs, leading to a reduction in neuronal excitability and representing the neuroprotective role of adenosine in excitotoxic events [15]. Considering the importance of adenosine and NMDA receptors in sleep processes [16,17] and the interrelationship between these two systems, we investigated whether there is a causal relationship between changes in A1R and synaptic NMDA receptors. The finding of significantly reduced levels of the A1R protein provides an explanation for the increased levels of proteins involved in glutamatergic signaling.

To our knowledge, this is the first study aimed at investigating the molecular changes in the components of the GABAergic and glutamatergic neurotransmitter systems in the hippocampus of adult male rats following zaleplon treatment within the therapeutic dose. Interestingly, the results have raised a new question regarding the involvement of the adenosine system in the mechanism of action of zaleplon.

## 2. Materials and Methods

All experimental procedures using laboratory animals were approved by the Ethics Committee for the Use of Laboratory Animals of the Vinca Institute of Nuclear Sciences—National Institute of the Republic of Serbia, University of Belgrade, Belgrade, Republic of Serbia (license number: 323-07-06,688/2020-05), according to the guidelines of the EU-registered Serbian Laboratory Animal Science Association (SLASA), a member of the Federation of the European Laboratory Animal Science Associations (FELASA). Care was taken to minimize pain and discomfort of the animals and all the experimental procedures were carried out in accordance with the UK Animals (Scientific Procedures) Act 1986, its associated guidelines, and the National Institutes of Health guide for the care and use of Laboratory animals (NIH Publications No. 80-23, revised 1996).

Adult male Wistar rats (2.5–3 months old) obtained from a local colony were housed under standard conditions: 12 h light/dark regime, ad libitum access to commercial rat pellets and tap water, and constant ambient temperature (21 ± 2 °C) and humidity. At the beginning of the experiment, the rats were randomly divided into two groups. In the first group, the rats were injected with the selected dose of zaleplon (Zal, *n* = 9 per group, 0.625 mg/kg *i. p.*), while the rats in the second group received the corresponding amount of saline (Con, *n* = 9 per group, vehicle *i. p.*) for five consecutive days. The zaleplon dose was chosen based on the acute dose-dependent effects, i.e., 0.625 mg/kg facilitated retrieval-based learning and produced anxiolytic-like effects without inducing sedation, as estimated in the active avoidance (AA) paradigm [18].

Upon completion of the zaleplon treatment, the rats were decapitated with a small animal guillotine (Harvard Apparatus, Holliston, MA, USA), and the brains were isolated for preparation of hippocampal synaptosomes (*n* = 6 per group) or immunohistochemical analysis (*n* = 3 per group). The hippocampus was selected as a target brain structure due to the following reasons: (1) high densities of GABA_A_ receptors [19], (2) its critical role in sleep-related memory processes [20], and (3) its contribution to the misuse, dependence, and withdrawal syndrome in patients using GABA_A_ receptor-positive modulators (although rare, as mentioned earlier) [21]. The isolated hippocampi were placed in an ice-cold isolation medium (0.32 M sucrose, 5 mM Tris-HCl, pH 7.4) for immediate preparation of synaptosomes, as described previously [22]. Briefly, after an initial short 10 min centrifugation at 1000× *g*, the resulting supernatant was transferred and centrifuged at 10,000× *g* for 20 min. The crude synaptosomal pellet was layered onto a Ficoll gradient, and the synaptosomes were collected after centrifugation at 65,000× *g* at 4 °C for 55 min. The protein content was determined with the modified Lowry method [23] using bovine serum albumin as a standard.

**Figure 1:**
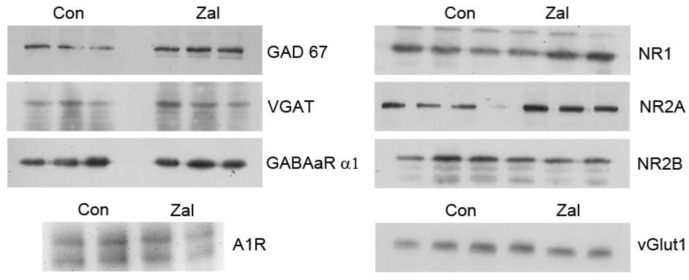
Representative Western blot analysis for the synaptosomal components of GABAergic and glutamatergic transmission and the adenosine A1R receptor. GABA_A_R α1—α1 subunit of the GABA_A_ receptor; vGlut1—vesicular glutamate transporter 1; NR1, NR2A, NR2B—subunits of the N-methyl-d-aspartate (NMDA) glutamate receptor; GAD67—67 kDa isoform of glutamate decarboxylase; VGAT—vesicular GABA transporter; A1R—adenosine receptor; and β-actin—beta actin.

After determining the protein concentration, an equal amount of total proteins from each sample was diluted in Laemmli sample buffer (250 mM Tris–HCl, pH 6.8, 10% SDS, 30% glycerol, 5% β-mercaptoethanol, 0.02% bromophenol blue), separated on 8% or 10% of SDS-PAGE gel, depending on the molecular weight of the target protein, and transferred to PVDF (polyvinylidene difluoride) membranes (Imobilion-P membrane, Millipore, Billerica, MA, USA). The membranes were blocked in TBS with 5% of non-fat milk (Sigma-Aldrich, Burlington, MA, USA) and 0.1% of Tween 20 (Sigma-Aldrich, Burlington, MA, USA) for 1 h, incubated with a primary antibody overnight at 4 °C, followed by incubation with a horseradish peroxidase-conjugated secondary antibody for 1 h at room temperature (RT) (Table 1). After washing in TBST, the membranes were incubated with the enhanced chemiluminescence system (Immobilon Western Chemiluminescent HRP Substrate, Millipore, Billerica, MA, USA), and immunoreactive bands were detected on X-ray films in a dark chamber or using the ChemiDoc-It^2^ 510 Imaging System. The signal intensity was evaluated using the Image J software (https://imagej.net/ij/index.html), accessed on 21 December 2022, with β-actin as a loading control. Representative Western blot images can be found in Figure 1.

The Western blot data were presented as the mean (% of control) ± SEM, and a two-tailed Student’s t test was used to compare the means between two groups. An alpha level of 0.05 was considered significant in the statistical test.

For an immunohistochemical analysis, the isolated brains were fixed in 4% paraformaldehyde (dissolved in 0.1 M PBS) for 24 h at 4 °C. After fixation, the brains were transferred into graded sucrose (10–30% in 0.2 M phosphate buffer) for cryoprotection at 4 °C. The dorsal hippocampus (−3.12 to −3.84 mm AP to Bregma) was cryosectioned in 25 µm thick coronal sections, air-dried for 2 h at RT, and stored at −20 °C until further use.

The sections were first hydrated at 37 °C for 30 min in 1× PBS and then permeabilized using Triton (0.5%) for 15 min. Furthermore, the sections were blocked in 1× PBST with 0.1% of Triton and 10% of goat serum for 1 h at RT and incubated overnight at 4 °C with a rabbit polyclonal anti-GABA (1:500, A2062 Sigma-Aldrich, Burlington, MA, United States) primary antibody. The sections were subsequently washed in PBST and incubated with an anti-rabbit secondary antibody (1:400 dilution in PBS, Alexa 488, Invitrogen, Waltham, MA, United States) for 2 h at RT. After washing in PBS, the slides were covered with a DAPI mounting medium (Sigma-Aldrich, Burlington, MA, USA) and evaluated using fluorescent microscopy. Micrographs were captured on an Axio Observer Microscope (Z1 AxioVision 4.6 software system, Carl Zeiss, Oberkochen, Baden-Wurttemberg, Germany) at 20× magnification. The presented microscopic images are representative findings obtained from two separate stainings for each group (*n* = 3). In each animal, five fields from two to four nonadjacent hippocampal sections were analyzed.

## 3. Results

**Figure 2:**
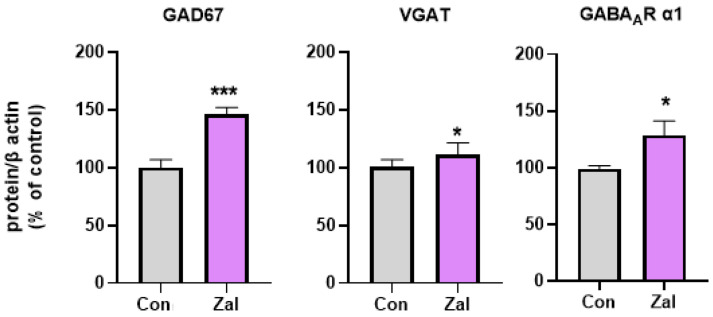
Protein levels of components of the GABAergic systems after prolonged treatment with 0.625 mg/kg zaleplon (Zal). Asterisks indicate a significant increase in the studied parameters in the Zal group compared to the Con group (* *p* < 0.05, *** *p* < 0.001). GABA_A_R α1—α1 subunit of the GABA_A_ receptor; vGlut1—vesicular glutamate transporter 1; GAD67—67 kDa isoform of glutamate decarboxylase; VGAT—vesicular GABA transporter; and β-actin—beta actin.

The statistical analysis revealed a significant increase or trend towards higher hippocampal protein levels in the components of the GABAergic system. A significantly higher expression of GAD67 (t (9) = 5.1140, *p* = 0.00063, Supplementary Figure S1) was observed in the zaleplon-treated rats compared to the control group. Concerning the α1 subunit of the GABA_A_ receptor and the VGAT protein, the statistics revealed a trend toward increased levels of these proteins compared to the control rats ((t (10) = 2.2190, *p* = 0.050) and (t (8) = 2.2657, *p* = 0.053, respectively), Supplementary Figure S1). These findings suggest upregulatory effects of prolonged zaleplon treatment on several components of the GABAergic system in the rat hippocampus (Figure 2, Supplementary Figure S1).

**Figure 3:**
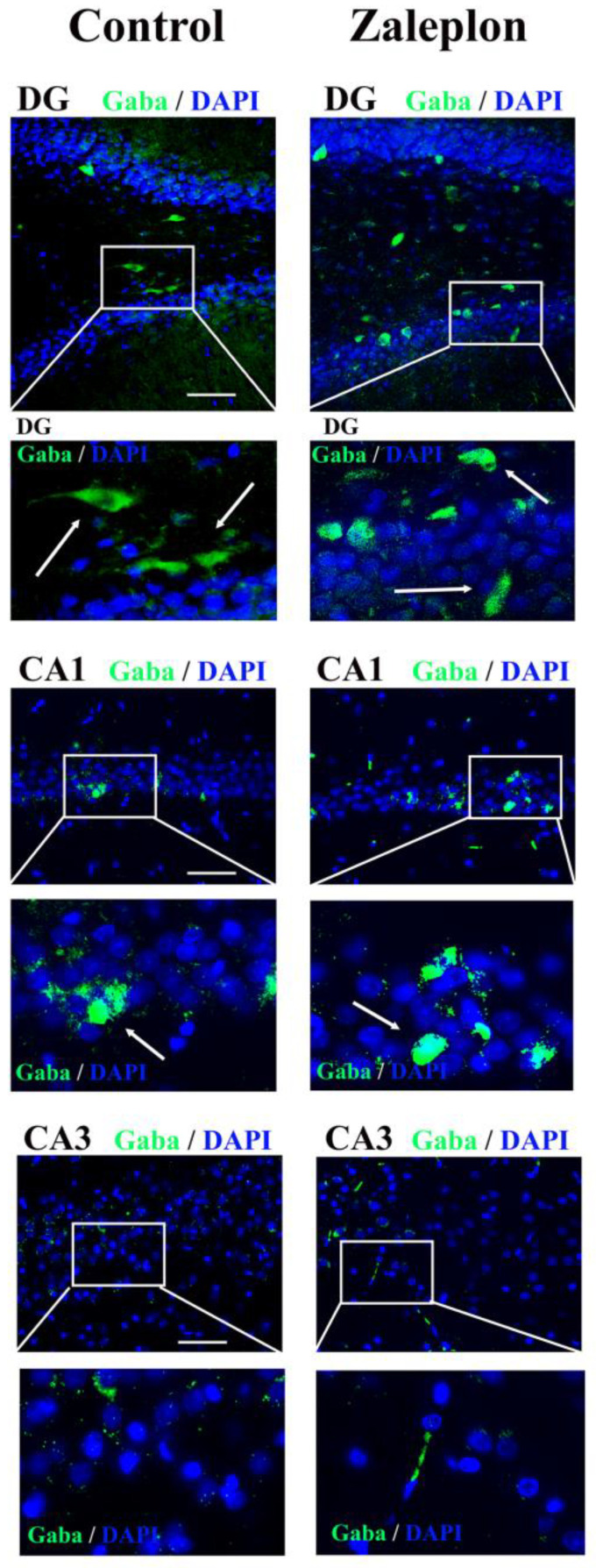
Immunohistochemical staining of the cryosectioned brains of rats treated with saline and 0.625 mg/kg zaleplon with anti-GABA antibody (green) in the dentate gyrus (DG) region and the CA1 and CA3 regions of the hippocampus. The cell nuclei were stained with DAPI (blue). GABA+ cells are marked with arrows.

Since the Western blot analysis revealed the increased expression of GAD67 and VGAT proteins in the synaptosomal fraction, immunohistochemical staining was performed to investigate the localization of GABA+ cells in the dorsal hippocampus (involved in learning, memory, sleep, etc.) using an anti-GABA antibody. GABA+ cells (green) were observed in the DG, CA1, and CA3 regions. The treatment with zaleplon increased the expression of GABA in the DG and CA1 regions, while GABA expression in the CA3 region remained unchanged (Figure 3). However, despite the significant differences in GABA expression in the specific hippocampal regions between the control and zaleplon–treated rats, the statistical analysis could not confirm significance due to the small number of samples (*n* = 3).

**Figure 4:**
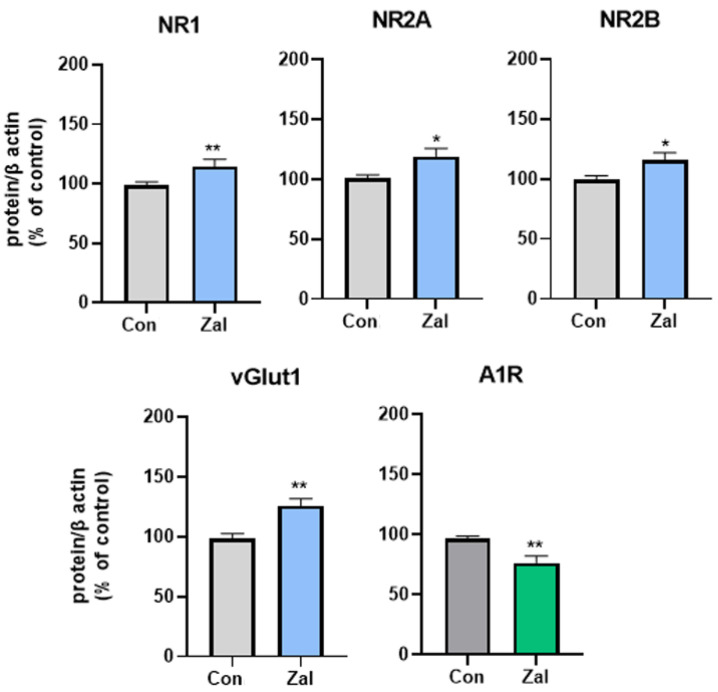
Protein levels of the components of the glutamatergic and adenosine systems after prolonged treatment with 0.625 mg/kg zaleplon (Zal). Asterisks indicate a significant increase in the studied parameters in the Zal group compared to the Con group (* *p* < 0.05, ** *p* < 0.01). vGlut1—vesicular glutamate transporter 1; NR1, NR2A, NR2B—subunits of the N-methyl-d-aspartate (NMDA) glutamate receptor; A1R—adenosine receptor; and β-actin—beta actin.

The statistical analysis indicated a significant increase in all the studied components of the glutamatergic system in the zaleplon-treated rats compared to the control group: vGlut1 (t (10) = 3.6056, *p* = 0.0048), NR1 (t (9) = 3,9789, *p* = 0.0032), NR2A (t (10) = 2,4564, *p* = 0.034), and NR2B (t (10) = 2.3640, *p* = 0.0397) (Figure 4, Supplementary Figure S1). On the other hand, A1R protein expression was significantly decreased in the hippocampus of the rats treated with zaleplon compared to the control group (t (8) = 3.3703, *p* = 0.0098, Supplementary Figure S1).

## 4. Discussion

Although there are numerous studies on the pharmacokinetics and pharmacodynamics of Z drugs, relatively few studies have addressed the changes in the components underlying their mechanism of action. In this study, we report, for the first time, changes in the expression of synaptic proteins involved in GABAergic and glutamatergic signaling in the rat hippocampus after 5 days of treatment with zaleplon. Importantly, we point out the significant role of the adenosine receptor in the mechanism of action of zaleplon, reflecting the complexity of the interaction of neurotransmitter systems in the brain.

Zaleplon is primarily indicated for the treatment of acute and chronic insomnia, a disorder associated with low GABA levels or impaired GABAergic transmission [11,24,25]. Specifically, magnetic resonance spectroscopy revealed that the overall levels of GABA in the brain regions of patients with insomnia were lower than in the healthy controls [26,27,28]. A significant decrease in the GABA concentrations in the discrete brain regions was also associated with sleep deprivation in rats [29]. Consistent with the sedative–hypnotic mechanism of zaleplon action, the results of our study suggest increased GABA synthesis and its packaging into vesicles, as evidenced by higher levels of the GAD67 protein and GABA immunostaining and VGAT proteins, respectively. Previous studies reported increased levels of GABA in the whole brain and plasma of healthy rabbits after administration of both low and high doses of zaleplon [30], whereas the same effect was observed in the whole brain of rats at the high dose of zaleplon (4 mg/kg, p. o.) but not at the low dose (1 mg/kg, p. o.) [31]. In particular, our results distinguish the more intense immunohistochemical GABA staining in the DG and CA1 hippocampal regions of the zaleplon-treated rats. In addition, increased protein levels of the GABA_A_ receptor α1 subunit, which is responsible for the sedative effect of zaleplon, were detected. This finding could provide an explanation for the safe pharmacological profile of zaleplon, i.e., the rare development of side effects (tolerance, abuse, dependence, etc.) compared to, e.g., benzodiazepines, where prolonged treatment leads to a significant decrease in the α1 subunit of the GABA_A_ receptor, a key event for the occurrence of tolerance [19]. However, based on our results, it is not possible to draw a conclusion as to whether the increase in the number of the α1 subunit of GABA_A_ receptors is a consequence of de novo synthesis or only its relocation from the extracellular space. More specifically, synaptic GABA_A_ receptors are recruited directly from their extrasynaptic counterparts [32,33], revealing a dynamic mechanism by which neurons modulate the number of GABA_A_ receptors located at inhibitory synapses. However, since significantly lower mRNA levels of the α1 and α2 subunits of GABA_A_ receptors were found in the insomnia disorder group [11], we might speculate that zaleplon increases the expression of GABA_A_ receptor subunits that are downregulated in insomnia.

Glutamate, a primary excitatory neurotransmitter and an important intermediate of cell metabolism in the brain, has a far-reaching influence on the sleep−wake regulatory system [34]. Sleep deprivation has been associated with a significant increase in glutamate levels in the hippocampus [35], prefrontal cortex, and thalamus of rats [29]. In addition, several pathologies associated with insomnia are characterized by increased glutamate levels in different brain regions [36]. Surprisingly, we observed increased protein levels of vGlut1 and the NR1, NR2A, and NR2B subunits of the NMDA receptor following treatment with zaleplon. It is known that the hippocampus is a crucial site for learning and memory processes [37] and that the induction of long-term potentiation (LTP), an important cellular mechanism responsible for learning and memory, is typically dependent on the activation of NMDA glutamate receptors. The resulting increase in Ca^2+^ influx into postsynaptic cells activates a number of biochemical processes in these neurons, including the formation of nitric oxide (NO) [38]. The aforementioned glutamate—NMDA receptor—nitric oxide synthase—NO system plays an essential role in the modulation of learning-related synaptic plasticity such as learning-dependent LTP [39]. A previous study by Samardzic and their co-authors aimed to investigate the behavioral profile of acutely administered different doses of zaleplon in rats using the active avoidance test and the elevated plus maze. Their results suggest that very low doses of zaleplon facilitate retrieval-based learning and produce anxiolytic-like effects without causing sedation [18]. Given that we have demonstrated enhanced components of glutamatergic signaling and in light of the aforementioned role of the NMDA, it will be of interest to further investigate the behavioral consequences of zaleplon treatment and test the intriguing hypothesis of the memory-enhancing effects of zaleplon.

In the search for the underlying mechanism for the detected increase in glutamatergic components, adenosine has emerged. Adenosine can be considered a central excitatory and inhibitory neurotransmitter and an important sleep-regulating substance [40,41]. Adenosine promotes sleep induction via A1 receptors, whereas prolonged sleep deprivation has been associated with an upregulation of the A1 receptors in different regions of the human [42,43] and rat brain [44]. In addition, glutamate released from the presynaptic membrane is known to activate astrocytes, which, in turn, release ATP and adenosine [34]. These molecules bind to purinergic P2Y and adenosine A1 receptors, respectively, activating them and triggering heterosynaptic suppression [45]. In particular, the activation of A1 receptors leads to a combined effect of presynaptic inhibition of glutamate release and reduced activation and surface expression of postsynaptic NMDA receptors [16,34], thereby suppressing neuronal hyperexcitability. We were able to demonstrate significantly reduced protein levels of A1 receptors in the hippocampus following prolonged zaleplon administration. This evidence of significantly reduced A1 receptor protein levels may provide an explanation for the increased levels of the proteins involved in glutamatergic signaling and has raised a new question to which we are seeking an answer in our ongoing study—the involvement of the adenosine system in the mechanism of action of zaleplon.

## 5. Conclusions and Future Prospects

To our knowledge, these are the first results reporting molecular changes in the neurotransmitter systems in the hippocampus of naive adult male rats following zaleplon treatment within the therapeutic dose. Considering that zaleplon belongs to the group of hypnotics, our results showing an enhancement of GABAergic signaling by zaleplon are not surprising, as decreased GABA neurotransmission is associated with the insomnia disorder. Unexpectedly, however, we observed increased protein levels of components of glutamatergic signaling in the zaleplon-treated rats. This phenomenon could be explained by a downregulation of the adenosine A1 receptors in the hippocampus, an important modulator of the glutamatergic system, which was also detected after prolonged zaleplon administration. In our ongoing study, we will investigate the effects of the observed zaleplon-induced increase in glutamatergic neurotransmission in the hippocampus and the role of the adenosine system in the mechanism of action of zaleplon as well.

## 6. Authors’ Considerations and Limitations of the Study

The authors would like to emphasize that there has been very little to no research on molecular studies of Z drugs in general. Therefore, the data presented provide valuable and, to our knowledge, the first information on components of GABAergic and glutamatergic neurotransmission following zaleplon treatment and highlight the adenosine system as an important modulator of zaleplon’s mechanism of action.

The main objective of the study was to investigate the mechanism of action of zaleplon, focusing on relevant neurotransmitter systems. As therapeutic outcomes in pathological conditions were not the focus of the study, animal models of sleep disorders were not used, although it would certainly be relevant to investigate the effect of zaleplon in these pathological conditions. The use of the synaptosomal fraction also limits the conclusions that can be drawn about the proteins studied. Therefore, the inclusion of other tissue fractions in the future would provide more detailed data on the overall process.

## Figures and Tables

**Table 1:** List of primary and secondary antibodies used for the Western blot analysis.

Antigen	Manufacturer/Catalog No.	Species/Dilution
GABA_A_R α1	Sigma Aldrich, Burlington, MA, USA, G4416	rabbit polyclonal, 1:5000
vGlut1	Abcam, Cambridge, UK, 134283	mouse monoclonal, 1:4000
NR1	Cell Signaling Technology, Danvers, MA, USA, # 5704	rabbit polyclonal, 1:1000
NR2A	Merck Millipore, Burlington, MA, USA, # 07-632	rabbit polyclonal, 1:1000
NR2B	Abcam, Cambridge, UK, 93610	mouse monoclonal, 1:4000
GAD67	Millipore, Burlington, MA, MAB5406	mouse monoclonal, 1:1000
VGAT	Santa Cruz Biotechnology, Dallas, TX, USA, sc-393373	mouse monoclonal, 1:1000
A1R	Alomone labs, Jerusalem, Israel	rabbit polyclonal, 1:1000
β-actin	Thermo Fisher Scientific, Waltham, MA, USA; PA1-21167	rabbit polyclonal, 1:5000
mouse IgG	R&D systems, bio-techne, Minneapolis, MN, USA; HAF007	goat polyclonal, 1:10,000
rabbit IgG	Invitrogen, Waltham, MA, USA; # 31460	goat polyclonal, 1:10,000

***Note:*** GABA_A_R α1—α1 subunit of the GABA_A_ receptor; vGlut1—vesicular glutamate transporter 1; NR1, NR2A, NR2B—subunits of the N-methyl-d-aspartate (NMDA) glutamate receptor; GAD67—67 kDa isoform of glutamate decarboxylase; VGAT—vesicular GABA transporter; A1R—adenosine receptor; and β-actin—beta actin.

## Data Availability

The data are contained within the article.

## Supplementary Materials

The following supporting information can be downloaded at: https://www.mdpi.com/article/10.3390/brainsci13121707/s1, Figure S1: original (unprocessed) images of representative blots.
