# Supplementary material for: Prolonged Zaleplon Treatment Increases the Expression of Proteins Involved in GABAergic and Glutamatergic Signaling in the Rat Hippocampus
Source: Brain Sci. 2023 Dec 12;13(12):1707. doi: 10.3390/brainsci13121707 (PMC10741523; doi:10.3390/brainsci13121707)
Supplement: Supplementary file 1 [file brainsci-13-01707-s001.zip › brainsci-2751681-supplementary.pdf]

Original (unprocessed) images of representative blots

Note: Due to the different molecular weight of the proteins the membranes were cut (where possible) or stripped and reprobed for another protein.

Con Zal

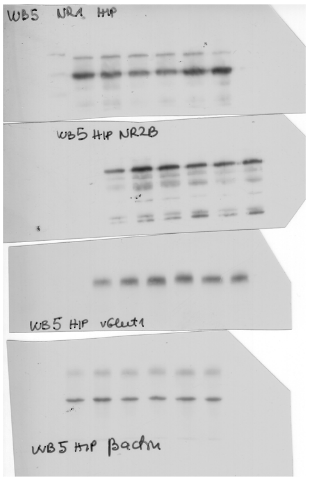

Con Zal

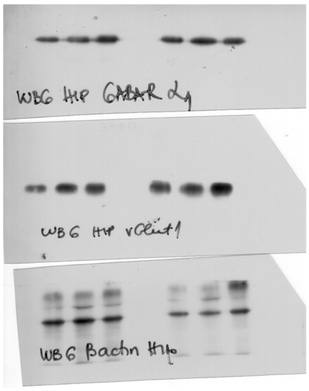

Con Zal

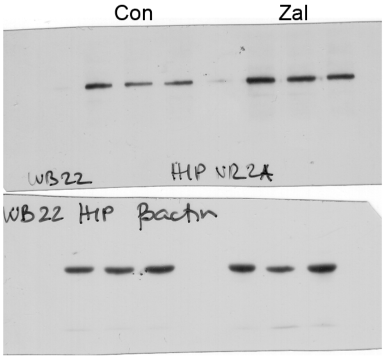

GAD 67

Con Zal

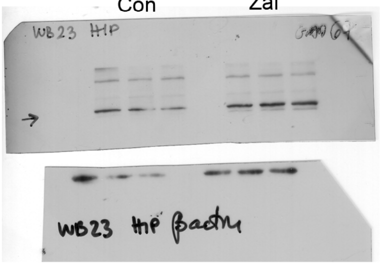

VGAT

Zal Con

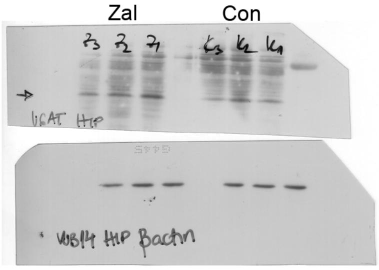

A1R

Con Zal Con Zal Con Zal

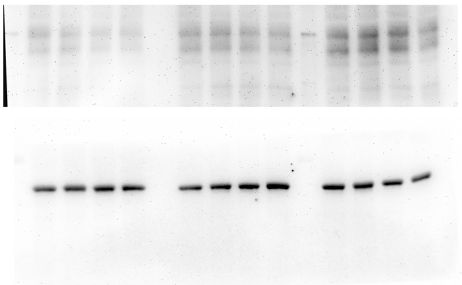

Special note: Unlike other blots, A1R chemiluminiscent signals were detected by ChemiDoc-It Imager (Ultra-Violet Products Ltd., Cambridge, United Kingdom)
